# It’s Never Too Late: A Case of Cystic Fibrosis Diagnosed at the Age of 58

**DOI:** 10.7759/cureus.92472

**Published:** 2025-09-16

**Authors:** Federica Di Giorgi, Miriam De Francesco, Annabella Catone, Marta Lomi

**Affiliations:** 1 Department of Surgical, Medical and Molecular Pathology and Critical Care Medicine, University of Pisa, Pisa, ITA

**Keywords:** 3849+10kbc>t mutation, bronchiectasis, cystic fibrosis, genetic therapy, pulmonary infections

## Abstract

Cystic fibrosis (CF) is rarely diagnosed in adulthood, but milder forms with residual cystic fibrosis transmembrane conductance regulator (CFTR) function can delay diagnosis. The 3849+10KbC>T mutation, a class V splicing mutation, results in residual CFTR function and atypical CF presentations, often remaining undiagnosed until adulthood. In these cases, neonatal screening may be negative. CF should be suspected in adults with bronchiectasis, especially when localized to the upper lobes or associated with nutritional deficiencies, recurrent pancreatitis, infertility, or nasal polyps. Early diagnosis and CFTR-targeted therapies can significantly improve disease outcomes and quality of life.

We present the case of a 58-year-old patient with delayed CF diagnosis due to atypical disease presentation, loss to follow-up, and pulmonologists' reluctance to consider CF in adult bronchiectasis. This case highlights the importance of thorough evaluation of bronchiectasis in adults to ensure timely diagnosis and appropriate management.

## Introduction

Cystic fibrosis (CF) is an autosomal recessive genetic disorder caused by mutations in the cystic fibrosis transmembrane conductance regulator (CFTR) gene located on the long arm of chromosome 7. CFTR mutations are classified into six classes, based on the function of the resulting protein. The most common mutation is Phe508del, accounting for approximately 70% of CF alleles worldwide. The CFTR protein is a channel for chloride ions across epithelial cell membranes; when it is defective or absent exocrine gland secretions become viscous, leading to obstruction, inflammation, and chronic infection. The clinical manifestations include bronchiectasis, recurrent pulmonary infections, chronic sinusitis with nasal polyposis, malnutrition, diabetes, azoospermia, and focal biliary cirrhosis [1].

CF is one of the most common genetic disorders in Caucasian populations, with an estimated incidence of one in 2,500 to one in 3,500 live births in Europe and North America [2]. Due to the high incidence of CF, newborn screening is routinely performed in many countries. This typically involves measuring serum trypsin levels, which are below the normal range in patients with pancreatic insufficiency, facilitating early diagnosis. However, patients with mutations resulting in CFTR residual function may not have pancreatic insufficiency, leading to false-negative screening results [3].

In cases of clinical suspicion or positive screening, sweat chloride testing and genetic analysis are required for definitive diagnosis. While the majority of CF diagnoses are made early in childhood due to screening programs and the presence of clinical symptoms, it is important to consider that adults, particularly those born before the introduction of neonatal screening or with mild disease phenotypes, may be diagnosed later in life. Data from the Italian Registry shows that the incidence of new diagnosis in adulthood was 18.2% in 2012-2017 and 12% in 2018 of new CF diagnoses [4].

Recent advances in the therapeutic landscape, particularly the introduction of CFTR modulator therapies, have significantly improved the prognosis for CF patients [5]. CFTR modulators include potentiators and correctors. Potentiators help the channel stay open longer, while correctors help the protein fold and reach the cell surface. They are more effective when used together.

Early diagnosis is important in order to slow the decline in lung function, treat nutritional deficits and improve both quality of life and prognosis. 

We present a case of delayed diagnosis of CF, highlighting how the disease can remain undiagnosed despite the availability of diagnostic tools due to a lack of awareness of the disease in adulthood. We discuss the clinical and genetic characteristics that ultimately led to its identification in adulthood.

## Case presentation

The patient is a 58-year-old man, a former light smoker, with no family history of lung disease and two daughters in apparent good health. He had no significant pulmonary history during childhood. At the age of 14, during preoperative evaluation for nasal polypectomy, a chest X-ray revealed right pulmonary consolidation. Subsequent ventilatory-perfusion scintigraphy and hyperoxia testing led to the diagnosis of a pulmonary arteriovenous fistula.

**Figure 1:**
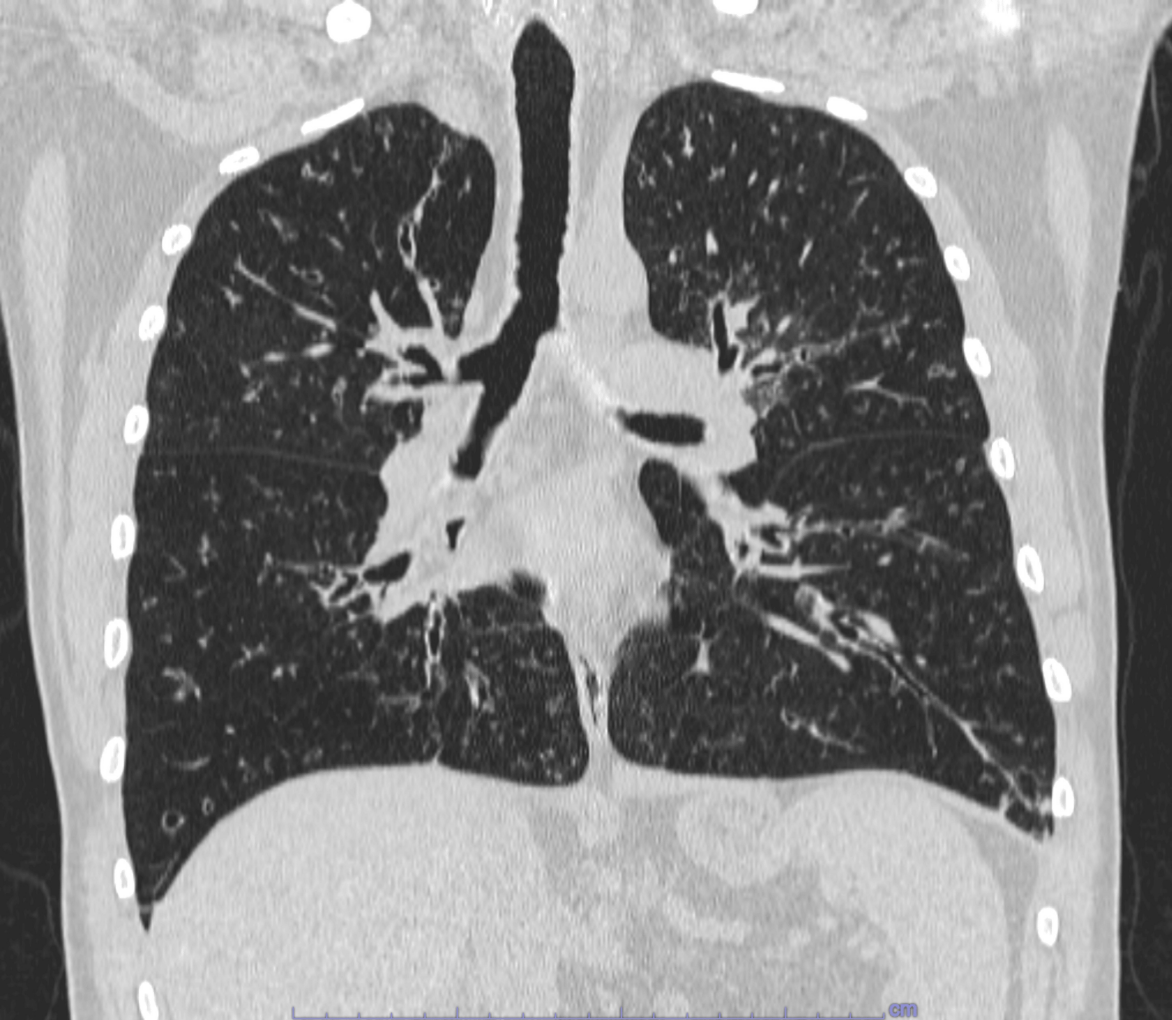
Chest CT scan performed in 2014 The CT shows bilateral cylindrical bronchiectasis and inflammatory involvement of the small airways

In 2014 (48 years old), he undertook pulmonary investigation due to the onset of worsening dyspnea, chronic cough, sputum production and frequent upper and lower respiratory tract infections in the previous years. Global spirometry revealed chronic obstructive pulmonary disease with severe obstruction: forced expiratory volume in the first second (FEV1) 0.84 L (26% of predicted value), forced vital capacity (FVC) 1.91 L (39% of predicted value), FEV1/FVC 0.44 (57% of predicted value). Chest CT showed diffuse cylindrical bronchiectasis (Figure 1).

Further testing excluded immune deficiency and alpha-1 antitrypsin deficiency. The patient was prescribed a combination of inhaled bronchodilator (long-acting beta-2 agonists and long-acting muscarinic antagonist) and corticosteroid therapy and long-term azithromycin.

Sputum cultures identified *Haemophilus parainfluenzae* since 2014 and *Staphylococcus aureus* since 2018. Unfortunately, the patient was lost to follow-up for eight years. However, during this period, the patient reported progressive dyspnea and frequent bronchial exacerbations that did not require hospitalization.

In February 2024, the patient was admitted, for the first time, to the intensive care unit for severe respiratory acidosis. At the time of admission, the patient presented with severe dyspnea, respiratory distress and sarcopenia (BMI 20.6 Kg/m 2 ). Blood gas analysis, performed with oxygen therapy at 3 L/min, showed pH 7.35, pCO2 83 mmHg, pO2 65 mmHg, and HCO3- 44,3 mmol/L. The patient was treated with noninvasive mechanical ventilation, empirical antibiotic therapy (piperacillin/tazobactam), methylprednisolone, and aerosol therapy (short-acting beta2-agonists and anticholinergics). 

**Figure 2:**
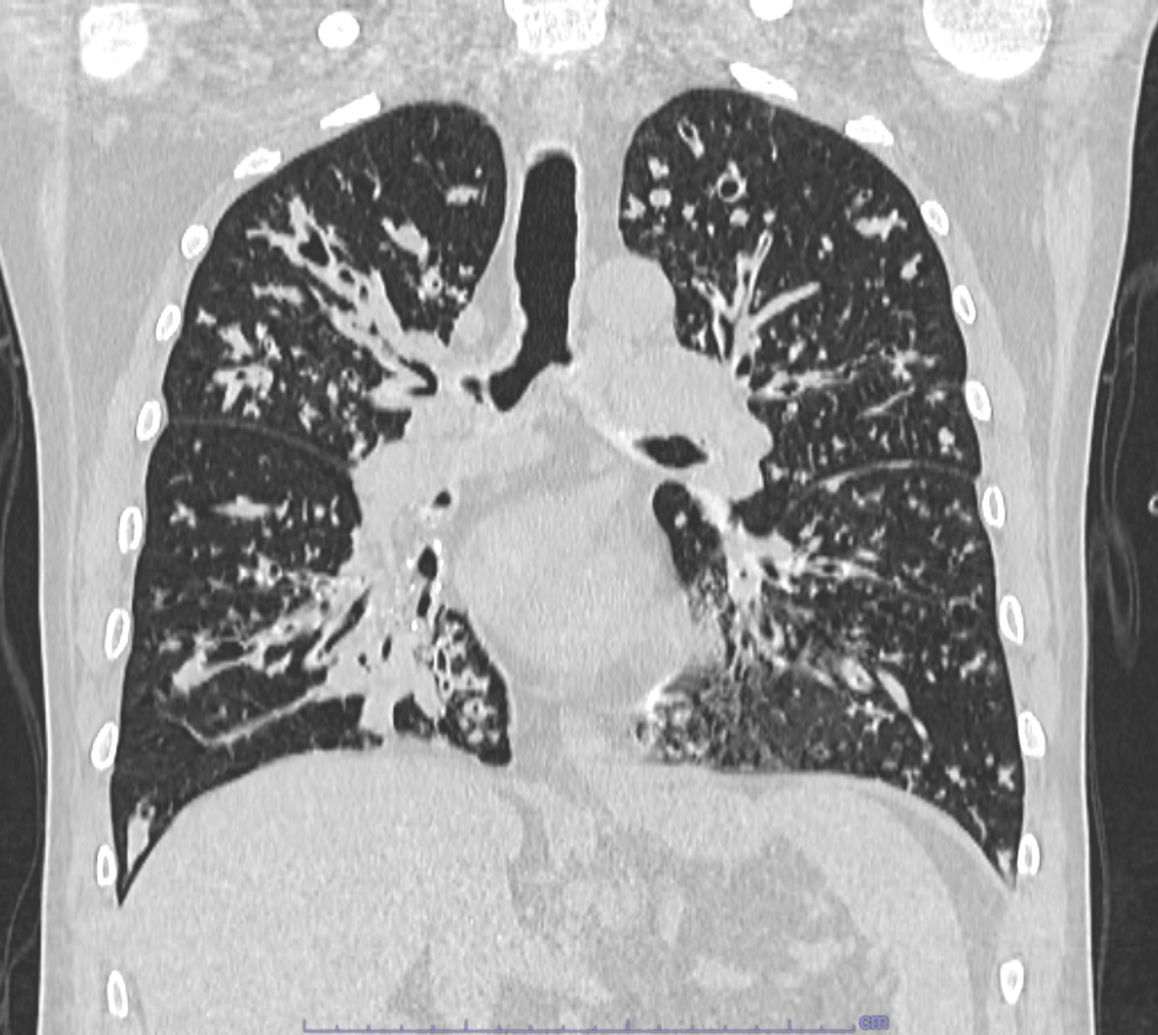
Chest CT scan performed in 2024 The CT scan shows progression of cylindrical and cystic bronchiectasis with predominant involvement of the upper lobes. Mucous infarcts of the bronchi and multiple peribronchial consolidations are also visible.

A chest CT revealed significant progression of bronchiectasis, particularly in the upper lobes (Figure 2).

Then, the patient was transferred to our sub-acute intensive care unit. *Pseudomonas aeruginosa* was detected in broncho-alveolar lavage by bronchoscopy, and the patient was treated with ceftolozane/tazobactam and levofloxacin. Long-term home noninvasive mechanical ventilation and oxygen therapy were initiated due to chronic hypercapnic respiratory failure. Additionally, he began respiratory physiotherapy using positive expiratory pressure devices.

During hospitalization, the diagnosis of CF was considered given the radiological progression, the localization of bronchiectasis in the upper lobes, the microbiological findings, and his teenage history. Genetic testing revealed the presence of two mutations: Phe508del and 3849+10KbC>T, confirming CF in compound heterozygosity. Sweat test was performed after the genetic diagnosis, due to technical reasons of our laboratory, with borderline results (chlorine levels in sweat 53 mEq/L). The patient was referred to a regional CF center, where he began targeted genetic therapy with ivacaftor, tezacaftor, and elexacaftor and alongside inhaled colistin. Fecal elastase was measured to assess exocrine pancreatic function. The result was 73.5 µg/g, indicating severe pancreatic insufficiency; therefore, enzyme replacement therapy was initiated. No further investigations into potential fertility issues were conducted.

The patient was also referred to a lung transplant center and is now on the waiting list for lung transplantation.

## Discussion

The 3849+10KbC>T mutation (also referred to as c.3718-2477C>T) is a class V splicing mutation, resulting in residual CFTR protein function, which may explain the atypical and delayed presentation of CF in this case [6]. This variant is associated with milder forms of CF that often go undiagnosed until adulthood [7,8].

In this case, the diagnosis of CF was delayed by nearly a decade from the first radiological finding of bronchiectasis. The delay in diagnosis can be attributed to the atypical presentation, which included adult-onset disease, apparent fertility, and absence of gastrointestinal symptoms. However, several factors should have raised suspicion, including the early pulmonary function impairment (FEV1 less than 1 liter before the age 50), the radiological progression, the microbiological findings of *Staphylococcus aureus* and *Pseudomonas aeruginosa*, and the patient’s history of adolescent nasal polyps.

In Italy, neonatal CF screening has been available since 1992, and most cases are detected through serum trypsin tests (not available for our patient). However, some CF forms, especially those with residual CFTR function, may not present with pancreatic insufficiency at birth and can thus evade detection by standard screening tests [3,9].

Therefore, CF should be considered in adults presenting with cystic bronchiectasis, particularly when the upper lobes are predominantly affected, and in those with a history of recurrent respiratory infections and no alternative explanation. Additional diagnostic clues include childhood nasal polyposis, as well as extrapulmonary comorbidities such as infertility, pancreatitis, and malnutrition [10,11], alongside typical microbiological findings of *Staphylococcus aureus* and *Pseudomonas aeruginosa* [12]. Although not pathognomonic, the presence of these diagnostic clues warrants the exclusion of CF, as it significantly impacts therapy and prognosis [13]. Even in adult patients, combination therapy with CFTR modulators (elexacaftor, tezacaftor, ivacaftor) has shown significant improvements in lung function, reduction in exacerbations, and improvement in nutritional status [14].

## Conclusions

CFTR mutations with residual protein function are associated with milder presentation of CF and are frequently undiagnosed until adulthood. CF should be considered in the differential diagnosis of adult patients with bronchiectasis, particularly when there is upper lobe predominance, a rapid decline in pulmonary function and microbiological isolation of *Pseudomonas aeruginosa* and Staphylococcus aureus. In this case sweat testing and/or genetic analysis should be performed. In advanced cases, referral to a transplant center is essential to ensure patients are evaluated for all available therapeutic options.

Early recognition of CF in adulthood can significantly alter the disease course, improving both clinical outcomes and quality of life, particularly with the advent of highly effective CFTR modulator therapies. In the era of precision medicine, where CFTR modulators exemplify targeted therapy, early diagnosis is the gateway to personalized and life-changing care.
